# Preoperative neutrophil to lymphocyte ratio and platelet to lymphocyte ratio are associated with major adverse cardiovascular and cerebrovascular events in coronary heart disease patients undergoing non-cardiac surgery

**DOI:** 10.1186/s12872-020-01500-6

**Published:** 2020-05-18

**Authors:** Jan Larmann, Jessica Handke, Anna S. Scholz, Sarah Dehne, Christoph Arens, Hans-Jörg Gillmann, Florian Uhle, Johann Motsch, Markus A. Weigand, Henrike Janssen

**Affiliations:** 1 Department of Anaesthesiology, Heidelberg University Hospital, Im Neuenheimer Feld 110, 69120 Heidelberg, Germany; 2 Department of Anaesthesiology and Intensive Care Medicine, Hannover Medical School, Hannover, Germany

**Keywords:** Coronary heart disease, Perioperative care, Blood cell count, Platelet to lymphocyte ratio, Neutrophil to lymphocyte ratio

## Abstract

**Background:**

Preoperative risk prediction in patients at elevated cardiovascular risk shows limited accuracy. Platelet to lymphocyte ratio (PLR) and neutrophil to lymphocyte ratio (NLR) indicate systemic inflammation. Both have been investigated for outcome prediction in the field of oncology and cardiovascular medicine, as well as risk prediction of adverse cardiovascular events in non-surgical patients at increased cardiovascular risk.

**Methods:**

For this post-hoc analysis, we included all 38 coronary heart disease patients from the Leukocytes and Cardiovascular Perioperative Events cohort-1 study scheduled for elective non-cardiac surgery. We evaluated preoperative differential blood counts for association with major adverse cardiovascular and cerebrovascular events (MACCE) defined as the composite endpoint of death, myocardial ischemia, myocardial infarction, myocardial injury after non-cardiac surgery, or embolic or thrombotic stroke within 30 days after surgery. We used Youden’s index to calculate cut-off values for PLR and NLR. Additive risk-predictive values were assessed using receiver operating characteristic curve and net reclassification (NRI) improvement analyses.

**Results:**

Patients with the composite endpoint MACCE had higher PLR and NLR (309 [206; 380] vs. 160 [132; 203], *p* = 0.001; 4.9 [3.5; 8.1] vs. 2.6 [2.2; 3.4]), *p* = 0.001). Calculated cut-offs for PLR > 204.4 and NLR > 3.1 were associated with increased risk of 30-day MACCE (OR 7, 95% CI [1.2; 44.7], *p* = 0.034; OR 36, 95% CI [1.8; 686.6], *p* = 0.001). Furthermore, NLR improved risk prediction in coronary heart disease patients undergoing non-cardiac surgery when combined with hs-cTnT or NT-proBNP (NRI _total_ = 0.23, *p* = 0.008, NRI _total_ = 0.26, *p* = 0.005).

**Conclusions:**

Both PLR and NLR were associated with perioperative cardiovascular adverse events in coronary heart disease patients. NLR proved to be of additional value for preoperative risk stratification. Both PLR and NLR could be used as inexpensive and broadly available tools for perioperative risk assessment.

**Trial registration:**

NCT02874508, August 22, 2016.

## Background

Indices between cell populations from differential blood count (DBC), which is often routinely performed in the clinical setting, are a valuable option to gain further information on systemic inflammation and have been of interest within different fields of medical research. Risk prediction by the neutrophil to lymphocyte ratio (NLR) and platelet to lymphocyte ratio (PLR) was initially studied in the field of oncology [1, 2], followed by a vast amount of studies on the predictive value of NLR and PLR in cardiovascular medicine [3–5] and in a general population over the age of 45 [6].

In recent years, investigators have also focused on the perioperative period as well. Both indices are associated with postoperative acute kidney injury [7, 8]. Elevated preoperative NLR is associated with mortality and adverse outcome after cardiovascular surgery [9, 10], as well as with myocardial injury after non-cardiac surgery [11, 12] and lower long-term remission of type 2 diabetes after metabolic surgery [13]. PLR is associated with all-cause complications after abdominal aortic aneurysm repair [14].

However, there is only limited data on the predictive value for cardiovascular events in the large group on non-cardiac surgery patients. First, it is unknown whether elevated PLR is associated with cardiovascular adverse events in non-cardiac surgery patients. Second, neither NLR nor PLR have been evaluated in coronary heart disease-patients undergoing non-cardiac surgery who face higher rates of perioperative cardiovascular complications with challenging and inferior risk prediction [15–18]. Third,it is unknown if NLR or PLR have additive value for biomarker based preoperative prediction of perioperative cardiovascular events.

Therefore, in this post-hoc analysis, we investigated if preoperative cell subset counts from a conventional DBC, as well as PLR or NLR, are associated with the composite endpoint major adverse cardiovascular and cerebrovascular events (MACCE) within the first 30 postoperative days in coronary heart disease patients undergoing non-cardiac surgery. In addition, we tested the additive value for prediction of cardiovascular events in coronary heart disease patients undergoing non-cardiac surgery when conventional risk-prediction is complemented by NLR and PLR.

## Methods

### Study population

Data from the Leukocytes and Cardiovascular Perioperative Events cohort-1 (LeukoCAPE-1) study were used to conduct a post-hoc analysis. LeukoCAPE-1 was a single-centre, prospective, observational cohort study conducted at the Department of Anaesthesiology, University Hospital Heidelberg, Heidelberg, Germany [17]. In summary, a total of 44 coronary heart disease patients scheduled for elective, non-cardiac surgery was screened for eligibility. Of those, 40 patients were successfully enrolled into the study. During follow-up, two patients withdrew consent and were excluded, resulting in a final study population of 38 participants. The main clinical and demographical baseline characteristics are published elsewhere [18]. In brief, the majority of patients were male, over the age of 60 years and classified as American Society of Anaesthesiologists physical status (ASA) ≥3. Patients in the MACCE group more frequently suffered from preoperative diagnosed renal failure (Kidney Disease: Improving Global Outcomes stage ≥3) and atrial fibrillation. Perioperative medication reflected patients’ elevated cardiovascular risk profile. Patients who suffered MACCE took angiotensin converting enzyme inhibitors more frequently. The incidence of a history of percutaneous coronary intervention was 29% in the MACCE and 42% in the no MACCE group. 29% of patients in the MACCE group reported a history of myocardial infarction (MI) compared to 48% in the no MACCE group [18]. The trial was registered prior to patient enrolment at clinicaltrials.gov (NCT02874508, Date of registration: August 22, 2016). The study protocol conformed to the principles of the Declaration of Helsinki and the World Medical Association. After approval by the local Ethics Committee of the medical faculty of the Ruprecht-Karls University Heidelberg (S-351/2016, 4th August 2016), and after informed written consent, we enrolled consecutive general, vascular and urological surgery patients with documented coronary heart disease undergoing elective, in-patient, non-cardiac surgery between August and October 2016. We excluded patients younger than 18 years, pregnant or breastfeeding, and individuals with leukaemia, leukocytosis (> 10 nl l^− 1^), or leukopenia (< 4 nl l^− 1^). Further exclusion criteria were emergency surgery, history of organ transplantation or splenectomy, immunosuppression, chemotherapy, GM-CSF or cortisone treatment less than 14 days prior to surgery, an intraoperative dexamethasone administration, or the occurrence of myocardial ischemia, MI, embolic or thrombotic stroke, congestive heart failure or serious cardiac arrhythmia within the past 28 days before enrolment.

### Data collection and conventional risk assessment

After enrolment, we recorded previous cardiovascular and cerebrovascular events, demographic data, ASA classification, smoking status and current medication. A 12-lead electrocardiogram was recorded preoperatively. Conventional risk assessment was based on revised cardiac risk index. Patients with high-sensitive cardiac Troponin T (hs-cTnT) ≥14 ng l^− 1^ or N-terminal proB-type natriuretic peptide (NT-proBNP) ≥300 ng l^− 1^ were considered as at risk for perioperative MACCE [19].

### Sample collection and laboratory measurements

As previously stated, blood samples were collected in the operating room prior to skin incision in EDTA for DBC and heparin tubes for biomarkers (Sarstedt, Nümbrecht, Germany) [17]. Samples were sent to the central laboratory within 30 min, and analyses were performed according to standard operating procedures. NT-proBNP was measured preoperatively (Immulite, Siemens Healthcare Diagnostics, Erlangen, Germany); hs-cTnT was determined preoperatively and on postoperative days 1 to 3 (Cobas E4111, Roche Diagnostics, Mannheim, Germany). Automated DBC were performed in the central laboratory.

### Outcome analysis

MACCE was defined as the composite endpoint of death, myocardial ischemia, MI, MINS, or embolic or thrombotic stroke within 30 days after surgery. In the LeukoCAPE-1 study a total of 7 patients reached this definition of the composite endpoint MACCE [18].

MINS [20] was defined as any raise in postoperative hs-cTnT ≥50 ng l^− 1^ [21] judged due to myocardial ischemia. Raising hs-cTnT was defined as an increase of at least 50% from baseline [22]. Other secondary endpoints were individual components of the primary endpoint MACCE, new-onset atrial fibrillation, peripheral vascular occlusion and acute kidney injury. For outcome analysis, a postoperative 12-lead electrocardiogram was recorded on the third postoperative day, patient charts were screened and, if discharged prior to postoperative day 30, patients or their family doctors participated in a scripted telephone interview at the end of follow-up.

### Statistical analysis

Not all blood cells were normally distributed. The Mann-Whitney U-test was chosen to compare all quantitative outcome data because it does not require any distributional assumption. Continuous data are presented as median [interquartile ranges (IQR)], unless otherwise stated. Boxes mark IQRs, whiskers extent from the 5th to 95th percentiles, and outliers are plotted as individual data points. Patients were categorized for the occurrence of MACCE. Two-sided *p* values < 0.05 were considered significant. To account for multiple comparisons, statistical analyses of preoperative leukocyte subset counts from DBC, as well as PLR and NLR were adjusted according to Bonferroni (α < 0.05/8). If patients were discharged early, hs-cTnT data were imputed (last observation carried forward analysis). Receiver operating characteristic (ROC) analyses were performed to evaluate the discriminatory power of PLR and NLR in association with MACCE. The optimal threshold was calculated based on the maximized Youden index. Odds ratios (OR) [95% confidence intervals (CI)] were calculated using Woolf and Baptista-Pike method as appropriate. Outcomes for ORs were all entities of the composite endpoint MACCE (death, myocardial ischemia, MI, MINS, or embolic or thrombotic stroke within 30 days after surgery), as well as all other secondary outcomes (new-onset atrial fibrillation, peripheral vascular occlusion and acute kidney injury). PLR and NLR cut-offs were used as independent variables. Net reclassification improvement (NRI) was performed to compare reclassification benefits and accuracies of preoperative PLR and NLR in addition to hs-cTnT or NT-proBNP for association with MACCE. The sample size (*n* = 40) for the LeukoCAPE-1 study was initially calculated to test the association of different leukocyte population counts with a surgical intervention [17]. Data from all 38 patients was included in this post-hoc analysis.

IBM SPSS Statistics 24.0 (SPSS, Chicago, USA), MedCalc 16.8 (MedCalc Software, Ostende, Belgium) and Prism 7.02 (GraphPad Prism Software, Inc., San Diego, US) were used for statistical analyses.

## Results

### Preoperatively conducted differential blood counts and calculated ratios

**Fig. 1:**
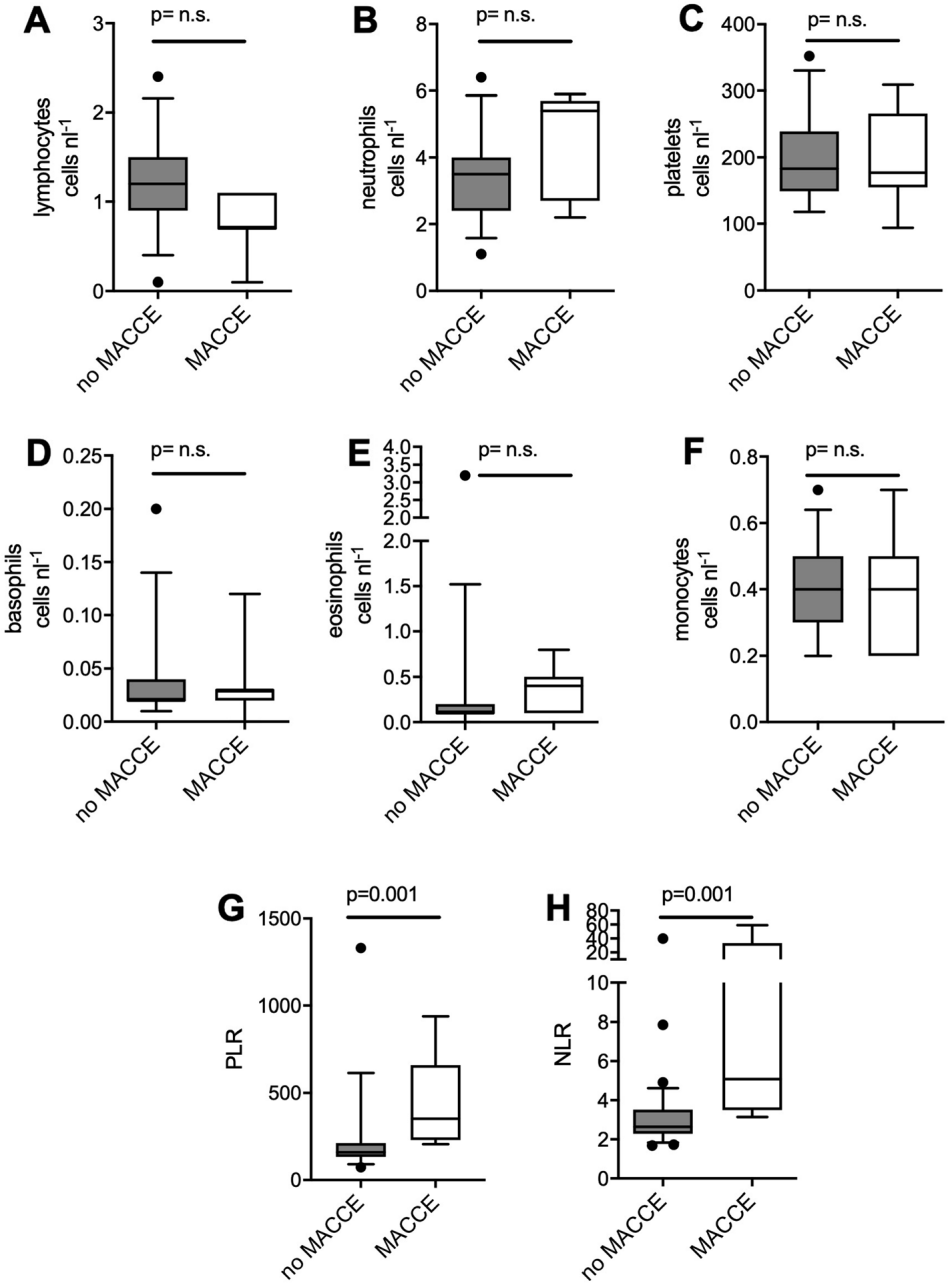
Differential blood count. Prior to non-cardiac surgery, differential blood counts were performed in the 38 included patients. **a-f** After adjustment for multiple comparisons (*p* = 0.05/8 = 0.006), no difference in subpopulations could be detected between patients who suffered MACCE and patients who did not. **g**, **h** Patients with MACCE had a higher PLR (309 [206; 380] vs. 160 [132; 203], *p* = 0.001), as well as higher NLR (4.9 [3.5; 8.1] vs. 2.6 [2.2; 3.4], *p* = 0.001) prior to surgery. MACCE: major adverse cardiovascular and cerebrovascular events, PLR: Platelet to Lymphocyte Ratio, NLR: Neutrophil to Lymphocyte Ratio

Results from DBC were recorded prospectively. Post-hoc, we calculated patients’ PLR and NLR after stratification for MACCE. After adjustment for multiple comparisons (Bonferroni (α < 0.05/8 = 0.006)), numbers of leukocyte subpopulations and platelets did not differ (Fig. 1 a-f). Patients suffering MACCE had higher PLR (309 [206; 380] vs. 160 [132; 203], *p* = 0.001) (Fig. 1 g) and higher NLR than patients without MACCE (4.9 [3.5; 8.1] vs. 2.6 [2.2; 3.4], p = 0.001 for MACCE vs. no MACCE) (Fig. 1 h).

### Receiver operating characteristics analysis of NLR and PLR

**Fig. 2:**
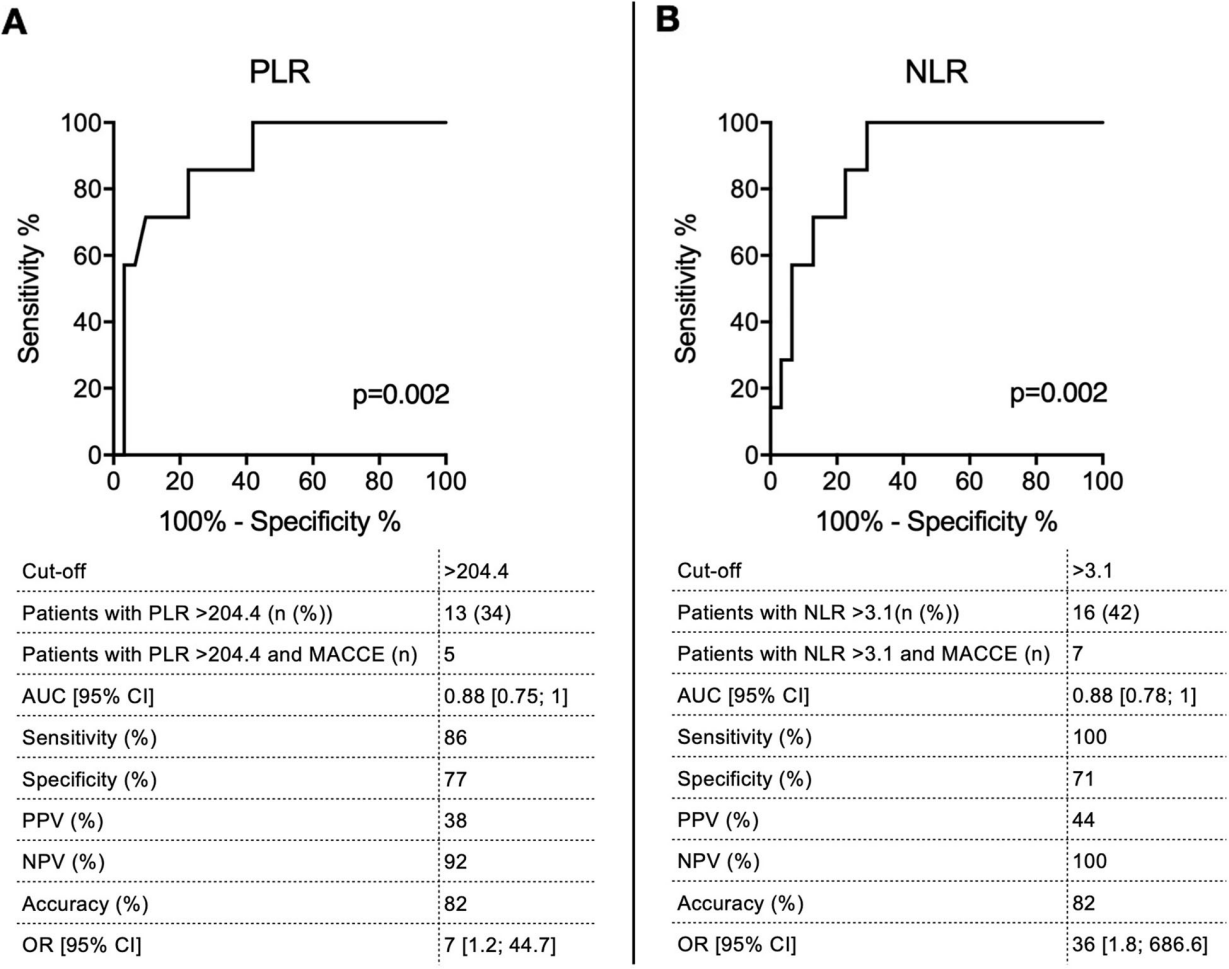
Receiver operating characteristics analysis of NLR and PLR. **a**, **b**) Receiver operating characteristics curve analyses were performed to evaluate discriminatory power of PLR and NLR for MACCE. Based on the maximized Youden Index, the optimal cut-off of PLR > 204.4 and NLR > 3.1 were calculated. Test characteristics based on Woolf Chi-square analyses are displayed. PLR: Platelet to Lymphocyte Ratio, NLR: Neutrophil to Lymphocyte Ratio, MACCE: major adverse cardiovascular and cerebrovascular events, AUC: Area under the curve, CI: confidence interval, PPV: positive predictive value, NPV: negative predictive value, OR: odds ratio

ROC curve analysis demonstrated that PLR had a high discriminatory value for MACCE (AUC = 0.88; 95% CI [0.75; 1], *p* = 0.002). Based on maximized Youden index, the optimal cut-off was calculated to be > 204.4 with a sensitivity of 86% and specificity of 77%. The accuracy of the test was 82% (Fig. 2 a). Also, preoperative NLR had a high discriminatory ability for MACCE in our cohort (AUC = 0.88; 95% CI [0.78; 1], p = 0.002). The optimal cut-off was calculated to be > 3.1 with a sensitivity of 100% and specificity of 71%. The accuracy of the test reached 82% (Fig. 2 b).

### Odds ratios for primary and secondary outcomes

**Fig. 3:**
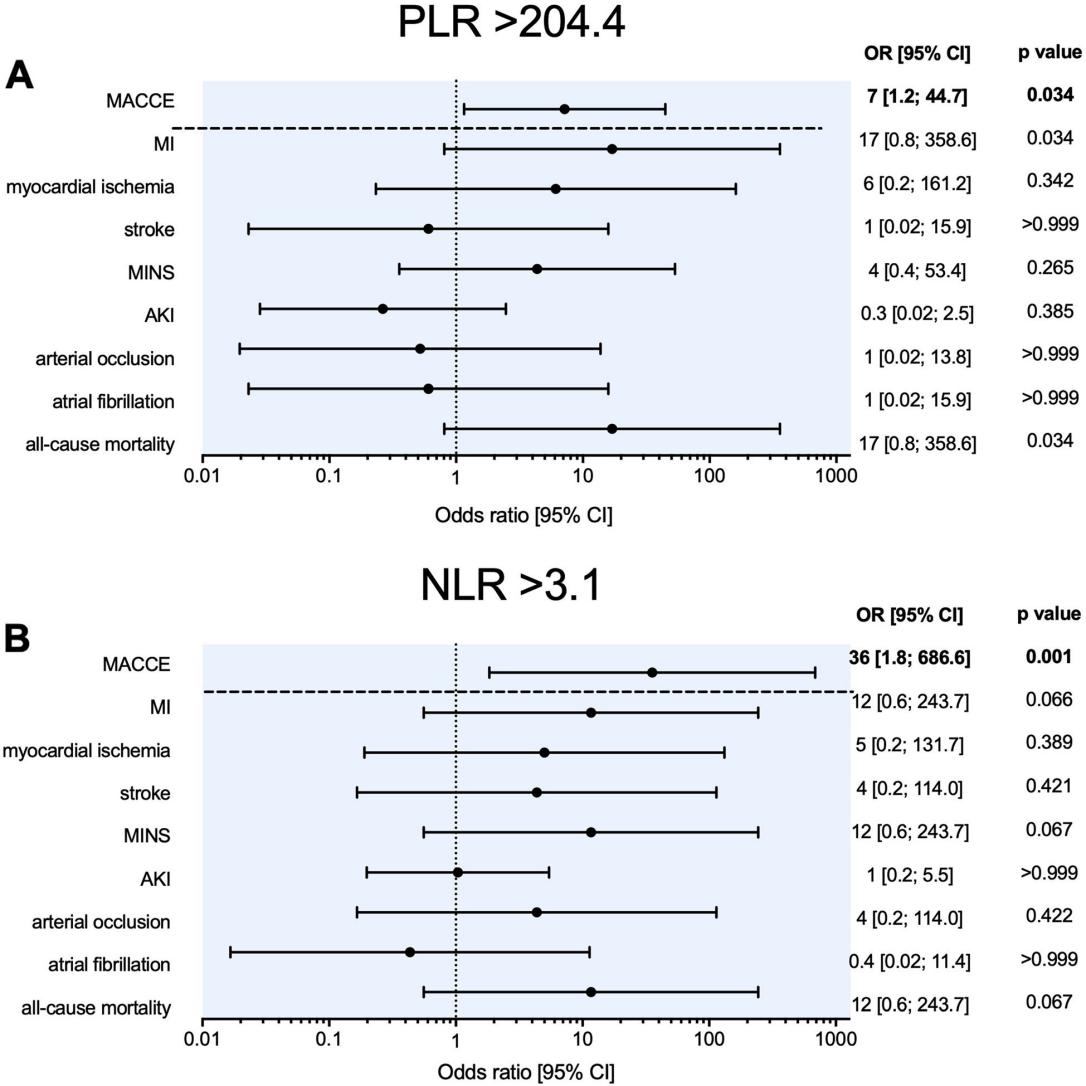
Odds ratios for MACCE and secondary outcomes. Woolf Chi-square analyses were performed to assess odds ratios. **a** A preoperative PLR of > 204.4 was associated with an increased risk of 30-day MACCE (OR 7; 95% CI [1.2; 44.7], *p* = 0.034. **b** A preoperative NLR > 3.1 was associated with an increased risk of 30-day MACCE (OR 36; 95% CI [1.8; 686.6], *p* = 0.001). In this population, no significant association was found for any of the secondary endpoints. PLR: Platelet to Lymphocyte Ratio, MACCE: major adverse cardiovascular and cerebrovascular events, MI: myocardial infarction, MINS: myocardial injury after non-cardiac surgery, AKI: acute kidney injury, OR: odds ratio, NLR: Neutrophil to Lymphocyte Ratio

A preoperative PLR of > 204.4 was associated with an increased risk of 30-day MACCE (OR = 7; 95% CI [1.2; 44.7], *p* = 0.034), but was not associated with any secondary outcome (Fig. 3 a). A preoperative NLR > 3.1 was associated with an increased risk of 30-day MACCE (OR = 36; 95% CI [1.8; 686.6], *p* = 0.001). There was no association with any secondary outcome (Fig. 3 b).

### Additive risk predictive value of PLR or NLR to hs-cTnT T or NT-proBNP

**Fig. 4:**
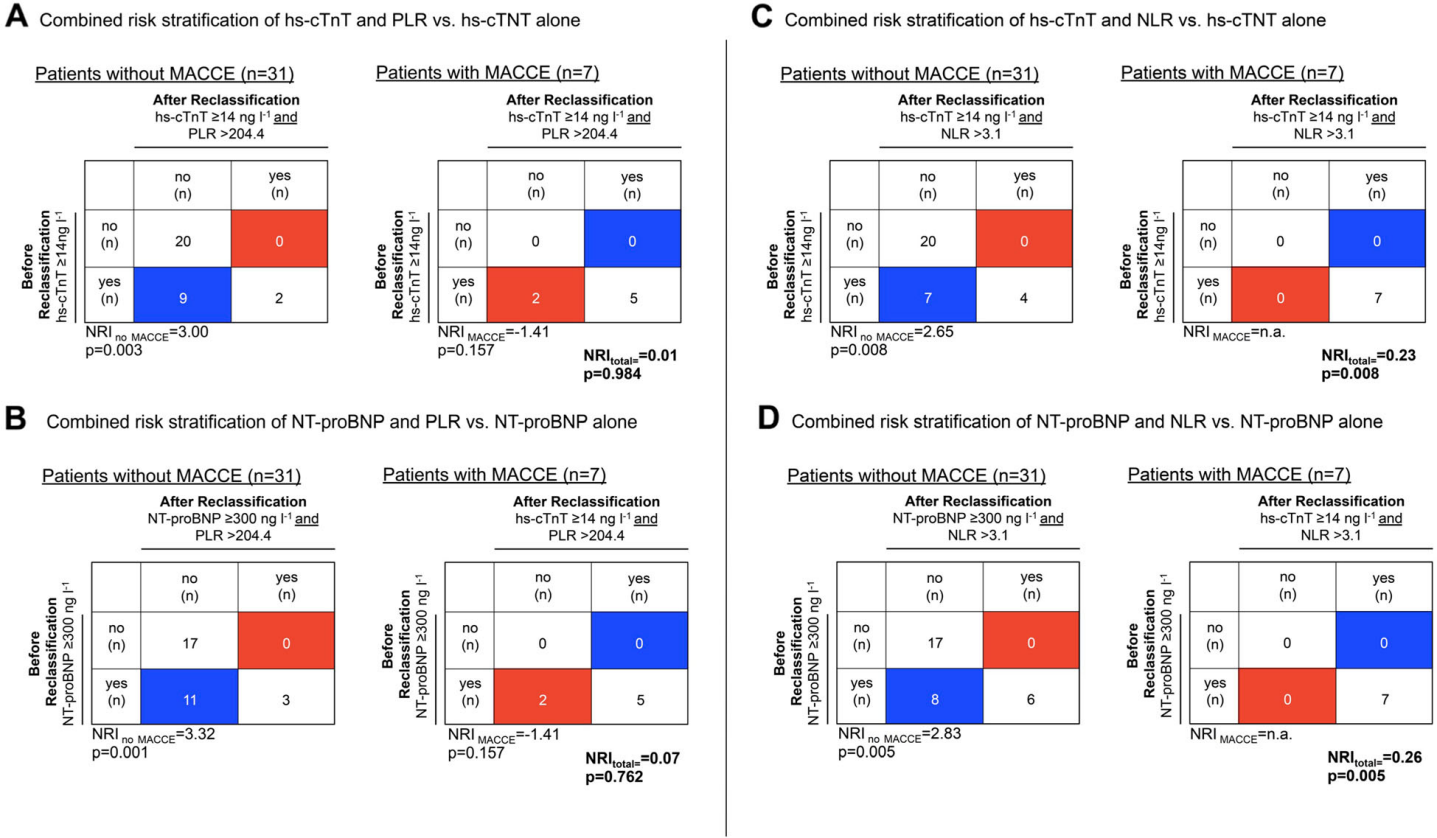
NET reclassification improvement analysis for NLR or PLR in addition to hs- cTnT or NT-proBNP for perioperative cardiovascular risk stratification. Reclassification tables are shown for patients with MACCE and patients without MACCE. yes: at risk, no: not at risk; Correctly reclassified patients: blue, incorrectly reclassified patients: red. **a**, **b** The combination of hs-cTnT or NT-proBNP with PLR > 204.4 did not show reclassification improvement (hs-cTnT: NRI_total_ = 0.01, *p* = 0.984; NT-proBNP: NRI_total_ = 0.07 *p* = 0.762). **c** The addition of NLR > 3.1 to hs-cTnT led to an overall improvement by seven correctly reclassified patients in the no MACCE group (NRI_total_ = 0.23, *p* = 0.008). **d** The addition of NLR > 3.1 to NT-proBNP led to the correct reclassification of eight patients in the no MACCE group and overall classification improvement (NRI_total_ = 0.26, *p* = 0.005). PLR: Platelet to Lymphocyte Ratio, NLR: Neutrophil to Lymphocyte Ratio, MACCE: major adverse cardiovascular and cerebrovascular events

Addition of PLR to hs-cTnT let to improvement in the group without MACCE by correct reclassification of nine patients in the “not at risk”-group (NRI _no MACCE_ = 3.00, *p* = 0.003). In the group with MACCE, two patients were wrongly reclassified in the “not at risk”-group (NRI _MACCE_ = − 1.41, *p* = 0.157). PLR did not prove to be of overall additional benefit for reclassification (NRI _total_ = 0.01, *p* = 0.984) (Fig. 4 a). Addition of PLR to NT-proBNP also let to improvement in the group without MACCE by reclassifying eleven patients (NRI _no MACCE_ = 3.32, *p* = 0.001). However, two patients were also wrongly reclassified in the “not at risk”-group (NRI _MACCE_ = − 1.41, *p* = 0.157). PLR did not prove to be of overall additional benefit for reclassification in combination with NT-proBNP (NRI _total_ = 0.07, *p* = 0.762) (Fig. 4 b). NRI analysis demonstrated that the combination of hs-cTnT and NLR improved risk stratification with overall increased reclassification accuracy (NRI _total_ = 0.23, *p* = 0.008) (Fig. 4 c). The addition of NLR to NT-proBNP also improved overall stratification (NRI _total_ = 0.26, *p* = 0.005) (Fig. 4 d). For both hs-cTnT and NT-pro-BNP-based risk stratification, NLR reduced false positive classifications in the group without MACCE (hs-cTnT: NRI _no MACCE_ = 2.65, *p* = 0.008; NT-proBNP: NRI _no MACCE_ = 2.83, *p* = 0.005) (Fig. 4 c, d).

## Discussion

In this post-hoc analysis of the LeukoCAPE-1 study, we show that high preoperative PLR and NLR exceeding calculated cut-offs might be associated with MACCE within 30 days after non-cardiac surgery in coronary heart disease patients. The addition of NLR to the established biomarkers hs-cTnT or NT-proBNP improved preoperative risk prediction for MACCE in this population. Leukocytes play a causal role in the pathogenesis of atherosclerosis and misbalances are part of the mechanisms of plaque progression, destabilization, erosion or rupture, leading to myocardial injury, MI and stroke [23].

After necessary adjustment for multiple comparisons, we could not detect any differences in blood cell subpopulations in our cohort other than in NLR and in PLR. Due to physiological and pathological changes of absolute counts, there is an argument for a higher stability of hematological indices [24]. Moreover, both PLR and NLR present as a ratio of two inversely directed mechanisms that exacerbate atherosclerosis and cardiovascular events [24]. In line with our finding that PLR is associated with adverse cardiovascular outcome after non-cardiac surgery in coronary heart disease patients, Azab et al. demonstrated that PLR is associated with mortality after MI, independently of lymphocyte or platelet counts [24]. We calculated an ideal cut-off for PLR at 204.4 based on Youden-index which was associated with MACCE. Compared to the recent literature, our cut-off ranks high. It has been shown that preoperative PLR > 153.6 predicts acute coronary syndrome in patients after carotid endarterectomy within an 18-month follow-up period [25], and extreme values of < 91.5 or > 163.3 are associated with complications after abdominal aortic aneurysm repair [26]. PLR was not associated with individual outcomes of the composite endpoint MACCE or any of the secondary outcomes analyzed in LeukoCAPE-1. As this analysis was conducted post-hoc, and the sample size was given, the study is underpowered to assess several secondary outcomes.

Our finding, that NLR is associated with adverse cardiovascular outcome, and improves risk prediction in coronary heart disease patients undergoing non-cardiac surgery, is in line with previous reports. In a cohort of patients at elevated cardiovascular risk, NLR is of predictive value for death or MI, even when DBC is within the normal range [27], and has proven to be a stronger independent predictor of mortality than other leukocyte subpopulations after MI [28]. NLR, but not DBC, predicts 2-year mortality after major vascular surgery [29]. Patients included in the LeukoCAPE-1 study underwent elective surgery. Correspondingly, median neutrophil count of 3.5 in our cohort is far lower than in patients undergoing emergency surgery [30], but also surprisingly low for patients with underlying chronic vascular inflammation [31].

Recently, two meta-anaylses have supported that high NLR on hospital admission of patients suffering MI is associated with deleterious outcome, including long-term mortality and adverse cardiovascular events [32, 33]. This extends to stroke, as high NLR is correlated with increased risk and worse functional outcome after ischemic stroke and with higher mortality after ischemic, as well as hemorrhagic stroke [34–36]. The majority of patients included in these studies have preexisting chronic cardiovascular disease. We could not detect any association with MI or stroke in our cohort, probably because the study is underpowered to test the secondary outcomes.

Patients with coronary heart disease are underrepresented in previous studies in the perioperative field [12]. Therefore, our cohort of patients with preoperatively diagnosed coronary heart disease is of particular interest for further studies into NLR as a predictive marker of cardiovascular events, especially as performance of current risk predicting tools is limited in this cohort [15]. Our post-hoc analysis showed that the inexpensive addition of NLR to the widely recommended biomarkers hs-cTnT or NT-proBNP [19, 37] improved risk classification mainly by reclassifying patients from “false positive” to “correct negative” which may lead to better resource management by reducing the number of patients stratified as at risk for MACCE.

Our study has several limitations. The analysis was performed post-hoc, rendering results explorative rather than confirmative. However, the results are hypothesis-generating and should be investigated in a larger prospective observational study in patients at elevated risk for perioperative cardiovascular complications. The number of patients is relatively low as it was originally calculated for testing the association of non-cardiac surgery with changes in counts of leukocyte subpopulation [17]. Confidence intervals for both NLR and PLR are wide and likely related to small sample size. However, the effect estimates not only for the primary endpoint MACCE, but also for other secondary cardiovascular endpoints such as MI, myocardial ischemia, MINS and all-cause mortality were ≥ 4 and can serve in the design of future clinical trials. Because of the limited number of patients, this study is insufficient for multivariate and logistic regression analyses. Future studies are needed to test whether NLR and PLR independently predict MACCE in patients at elevated risk for cardiovascular events.

## Conclusions

The findings of our post-hoc analysis suggest that both PLR > 204.4 and NLR > 3.1 are associated with perioperative 30-day MACCE in coronary heart disease patients. The addition of NLR to established biomarkers for cardiovascular risk stratification improved prediction in patients at elevated risk, potentially leading to improved resource management in perioperative care. PLR, as well as NLR are inexpensive, readily available markers. Preoperative PLR and NLR might be calculated to allow perioperative caregivers a more accurate prediction of perioperative cardiovascular events in coronary heart disease patients undergoing non-cardiac surgery.

## Data Availability

The datasets used and/or analyzed during the current study are available from the corresponding author on reasonable request.

## Abbreviations

ASA: American society of anaesthesiologists physical status
CI: Confidence interval
DBC: Differential blood count
Hs-cTnT: High-sensitive cardiac troponin T
IQR: Interquartile ranges
LeukoCAPE-1: Leukocytes and Cardiovascular Perioperative Events cohort-1
MACCE: Major adverse cardiovascular and cerebrovascular events
MI: Myocardial infarction
MINS: Myocardial injury after non-cardiac surgery
NLR: Neutrophil to lymphocyte ratio
NRI: Net reclassification improvement
NT-proBNP: N-terminal proB-type natriuretic peptide
OR: Odds ratio
PLR: Platelet to lymphocyte ratio
ROC: Receiver operating characteristic

## References

[1] Templeton AJ, McNamara MG, Seruga B, Vera-Badillo FE, Aneja P, Ocana A (2014). Prognostic role of neutrophil-to-lymphocyte ratio in solid tumors: a systematic review and meta-analysis. J Natl Cancer Inst.

[2] Li B, Zhou P, Liu Y, Wei H, Yang X, Chen T (2018). Platelet-to-lymphocyte ratio in advanced Cancer: review and meta-analysis. Clin Chim Acta.

[3] Balta S, Celik T, Mikhailidis DP, Ozturk C, Demirkol S, Aparci M (2016). The relation between atherosclerosis and the neutrophil-lymphocyte ratio. Clin Appl Thromb Hemost.

[4] Kalay N, Dogdu O, Koc F, Yarlioglues M, Ardic I, Akpek M (2012). Hematologic parameters and angiographic progression of coronary atherosclerosis. Angiology..

[5] Balta S, Ozturk C (2015). The platelet-lymphocyte ratio: a simple, inexpensive and rapid prognostic marker for cardiovascular events. Platelets..

[6] Fest J, Ruiter TR, Groot Koerkamp B, Rizopoulos D, Ikram MA, van Eijck CHJ (2019). The neutrophil-to-lymphocyte ratio is associated with mortality in the general population: the Rotterdam study. Eur J Epidemiol.

[7] Parlar H, Saskin H (2018). Are pre and postoperative platelet to lymphocyte ratio and neutrophil to lymphocyte ratio associated with early postoperative AKI following CABG?. Braz J Cardiovasc Surg.

[8] Chen D, Xiao D, Guo J, Chahan B, Wang Z. Neutrophil-lymphocyte count ratio as a diagnostic marker for acute kidney injury: a systematic review and meta-analysis. Clin Exp Nephrol. 2019. 10.1007/s10157-019-01800-y. 10.1007/s10157-019-01800-y 31650334

[9] Tan TP, Arekapudi A, Metha J, Prasad A, Venkatraghavan L (2015). Neutrophil-lymphocyte ratio as predictor of mortality and morbidity in cardiovascular surgery: a systematic review. ANZ J Surg.

[10] Silberman S, Abu-Yunis U, Tauber R, Shavit L, Grenader T, Fink D (2018). Neutrophil-lymphocyte ratio: prognostic impact in heart surgery. Early outcomes and late survival. Ann Thorac Surg.

[11] Ackland GL, Abbott TEF, Cain D, Edwards MR, Sultan P, Karmali SN (2019). Preoperative systemic inflammation and perioperative myocardial injury: prospective observational multicentre cohort study of patients undergoing non-cardiac surgery. Br J Anaesth.

[12] Durmus G, Belen E, Can MM (2018). Increased neutrophil to lymphocyte ratio predicts myocardial injury in patients undergoing non-cardiac surgery. Heart Lung.

[13] Bonaventura A, Liberale L, Carbone F, Vecchie A, Bonomi A, Scopinaro N (2019). Baseline neutrophil-to-lymphocyte ratio is associated with long-term T2D remission after metabolic surgery. Acta Diabetol.

[14] Lareyre F, Carboni J, Chikande J, Massiot N, Voury-Pons A, Umbdenstock E (2019). Association of Platelet to lymphocyte ratio and risk of 30-day postoperative complications in patients undergoing abdominal aortic surgical repair. Vasc Endovasc Surg.

[15] Ford MK, Beattie WS, Wijeysundera DN (2010). Systematic review: prediction of perioperative cardiac complications and mortality by the revised cardiac risk index. Ann Intern Med.

[16] Janssen H, Felgner L, Kummer L, Gillmann HJ, Schrimpf C, Rustum S (2020). Sequential surgical procedures in vascular surgery patients are associated with perioperative adverse cardiac events. Front Cardiovasc Med.

[17] Handke J, Scholz AS, Gillmann HJ, Janssen H, Dehne S, Arens C, et al. Elevated presepsin is associated with perioperative major adverse cardiovascular and cerebrovascular complications in elevated-risk patients undergoing noncardiac surgery: the leukocytes and cardiovascular perioperative events study. Anesth Analg. 2018. 10.1213/ANE.0000000000003738. 10.1213/ANE.0000000000003738 31094810

[18] Scholz AS, Handke J, Gillmann HJ, Zhang Q, Dehne S, Janssen H, et al. Frontline science: low regulatory T cells predict perioperative major adverse cardiovascular and cerebrovascular events after noncardiac surgery. J Leukoc Biol. 2019. 10.1002/JLB.5HI1018-392RR. 10.1002/JLB.5HI1018-392RR 31523852

[19] Duceppe E, Parlow J, MacDonald P, Lyons K, McMullen M, Srinathan S (2017). Canadian cardiovascular society guidelines on perioperative cardiac risk assessment and management for patients who undergo noncardiac surgery. Can J Cardiol.

[20] Botto F, Alonso-Coello P, Chan MT, Villar JC, Xavier D, Srinathan S (2014). Myocardial injury after noncardiac surgery: a large, international, prospective cohort study establishing diagnostic criteria, characteristics, predictors, and 30-day outcomes. Anesthesiology..

[21] Giannitsis E, Becker M, Kurz K, Hess G, Zdunek D, Katus HA (2010). High-sensitivity cardiac troponin T for early prediction of evolving non-ST-segment elevation myocardial infarction in patients with suspected acute coronary syndrome and negative troponin results on admission. Clin Chem.

[22] Alcock RF, Kouzios D, Naoum C, Hillis GS, Brieger DB (2012). Perioperative myocardial necrosis in patients at high cardiovascular risk undergoing elective non-cardiac surgery. Heart..

[23] Swirski FK, Nahrendorf M (2013). Leukocyte behavior in atherosclerosis, myocardial infarction, and heart failure. Science..

[24] Azab B, Shah N, Akerman M, McGinn JT (2012). Value of platelet/lymphocyte ratio as a predictor of all-cause mortality after non-ST-elevation myocardial infarction. J Thromb Thrombolysis.

[25] Bonaventura A, Carbone F, Liberale L, Mach F, Roth A, Burger F (2020). Platelet-to-lymphocyte ratio at the time of carotid endarterectomy is associated with acute coronary syndrome occurrence. J Cardiovasc Med (Hagerstown).

[26] Lareyre F, Carboni J, Chikande J, Massiot N, Voury-Pons A, Umbdenstock E, et al. Association of Platelet to lymphocyte ratio and risk of 30-day postoperative complications in patients undergoing abdominal aortic surgical repair. Vasc Endovascular Surg. 2019;53(1):5–11. 10.1177/1538574418789046 30021492

[27] Horne BD, Anderson JL, John JM, Weaver A, Bair TL, Jensen KR (2005). Which white blood cell subtypes predict increased cardiovascular risk?. J Am Coll Cardiol.

[28] Azab B, Zaher M, Weiserbs KF, Torbey E, Lacossiere K, Gaddam S (2010). Usefulness of neutrophil to lymphocyte ratio in predicting short- and long-term mortality after non-ST-elevation myocardial infarction. Am J Cardiol.

[29] Bhutta H, Agha R, Wong J, Tang TY, Wilson YG, Walsh SR (2011). Neutrophil-lymphocyte ratio predicts medium-term survival following elective major vascular surgery: a cross-sectional study. Vasc Endovasc Surg.

[30] Vaughan-Shaw PG, Rees JR, King AT (2012). Neutrophil lymphocyte ratio in outcome prediction after emergency abdominal surgery in the elderly. Int J Surg.

[31] Groot HE, van Blokland IV, Lipsic E, Karper JC, van der Harst P (2019). Leukocyte profiles across the cardiovascular disease continuum: a population-based cohort study. J Mol Cell Cardiol.

[32] Dentali F, Nigro O, Squizzato A, Gianni M, Zuretti F, Grandi AM (2018). Impact of neutrophils to lymphocytes ratio on major clinical outcomes in patients with acute coronary syndromes: a systematic review and meta-analysis of the literature. Int J Cardiol.

[33] Zhang S, Diao J, Qi C, Jin J, Li L, Gao X (2018). Predictive value of neutrophil to lymphocyte ratio in patients with acute ST segment elevation myocardial infarction after percutaneous coronary intervention: a meta-analysis. BMC Cardiovasc Disord.

[34] Song SY, Zhao XX, Rajah G, Hua C, Kang RJ, Han YP (2019). Clinical significance of baseline neutrophil-to-lymphocyte ratio in patients with ischemic stroke or hemorrhagic stroke: an updated meta-analysis. Front Neurol.

[35] Wang L, Song Q, Wang C, Wu S, Deng L, Li Y (2019). Neutrophil to lymphocyte ratio predicts poor outcomes after acute ischemic stroke: a cohort study and systematic review. J Neurol Sci.

[36] Giede-Jeppe A, Madzar D, Sembill JA, Sprugel MI, Atay S, Hoelter P, et al. Increased neutrophil-to-lymphocyte ratio is associated with unfavorable functional outcome in acute ischemic stroke. Neurocrit Care. 2019. 10.1007/s12028-019-00859-5. 10.1007/s12028-019-00859-5 31617117

[37] Kristensen SD, Knuuti J, Saraste A, Anker S, Botker HE, De Hert S (2014). 2014 ESC/ESA guidelines on non-cardiac surgery: cardiovascular assessment and management: the joint task force on non-cardiac surgery: cardiovascular assessment and management of the European Society of Cardiology (ESC) and the European Society of Anaesthesiology (ESA). Eur J Anaesthesiol.

